# Cronbach’s alpha in mathematics education research: Its appropriateness, overuse, and alternatives in estimating scale reliability

**DOI:** 10.3389/fpsyg.2022.1074430

**Published:** 2022-12-22

**Authors:** Yusuf F. Zakariya

**Affiliations:** Department of Mathematical Sciences, University of Agder, Kristiansand, Norway

**Keywords:** Cronbach’s alpha, reliability coefficient, internal consistency, unidimensionality, research instrument

## Abstract

Critiques of coefficient alpha as an estimate of scale reliability are widespread in the literature. However, the continuous overuse of this statistic in mathematics education research suggests a disconnection between theory and practice. As such, this article argues, in a non-technical way, for the limited usefulness of coefficient alpha, its overuse, and its alternatives in estimating scale reliability. Coefficient alpha gives information only about the degree of the interrelatedness of a set of items that measures a construct. Contrary to the widely circulated misconceptions in mathematics education research, a high coefficient alpha value does not mean the instrument is reliable, and it does not imply the instrument measures a single construct. Coefficient alpha can only be dependable as an estimate of reliability under verifiable and restrictive conditions. I expose these conditions and present steps for their verification in empirical studies. I discuss some alternatives to coefficient alpha with references to non-technical articles where worked examples and programming codes are available. I hope this exposition will influence the practices of mathematics education researchers regarding estimation of scale reliability.

## Introduction

The quality of studies that involve the measurement of constructs either in parts (as in mixed-methods research) or in a whole (as in quantitative research) is largely determined by the validity and reliability of the research instruments. Validity concerns the question of whether the instrument measures what it is purported to measure while reliability concerns the accuracy with which an instrument serves its purpose. The use of a scale that lacks either validity, reliability, or both in a study may water down the study to a mere play with numbers. This is because statistical analysis, interpretation of results, and subsequent implications of findings are substantially dependent on the level of validity and reliability of the instrument used for data generation. I mean, valid and reliable instruments raise one’s confidence in the generated data. Picking one at a time between validity and reliability, I delimit the scope of this article to issues around estimating scale reliability in mathematics education research.

Undoubtedly, Cronbach’s alpha coefficient (henceforth, coefficient alpha) is a well-received estimate of scale reliability among researchers in psychology, social sciences, health sciences, and education. At the time of writing this article, there were over 55900 Google scholar citations of the article in which Lee J. Cronbach popularized the coefficient. Over the last few decades, methodologists and researchers have made some critical comments that challenge the appropriateness of coefficient alpha for estimating scale reliability and proposed some alternatives in cases where coefficient alpha is not appropriate. Some researchers have called for total abandonment of coefficient alpha (e.g., Sijtsma, 2009; McNeish, 2018), some have recommended its continuous usage under strict and verifiable assumptions (e.g., Raykov, 1997; Raykov and Marcoulides, 2019), while others have either proposed or compared alternatives to coefficient alpha (e.g., Zumbo et al., 2007; Trizano-Hermosilla and Alvarado, 2016).

However, coefficient alpha is still widely used in mathematics education research, in many cases, as the only evidence of scale reliability. Apart from the popularity of the coefficient among mathematics education researchers, another concern is the prevalence of misapplications and misinterpretations of the coefficient some of which will be highlighted in this article. Could it be that mathematics education researchers are not aware of the problems with coefficient alpha? Perhaps, they are aware but are reluctant to change the practice. It could also be that the alternatives to coefficient alpha are not readily available to mathematics education researchers. Nevertheless, there is a need to open a discussion that highlights the appropriateness and overuse, and presents alternatives to coefficient alpha in estimating scale reliability in mathematics education research. This article sets to open such a discussion.

## Reliability of an instrument

Whenever we administer an instrument to measure a construct, we only have access to the respondents’ score called the observed score (x) on each item of the instrument. According to the classical test theory (Zimmerman, 1975; McDonald, 2011), the observed score, *x*, of an item can be decomposed into two unobserved (latent) scores: true score (*t*) and measurement error (*e*), i.e., *x* = *t* + *e*. Intuitively, the true score is the actual score of interest in measuring a construct while the measurement error is an inherent pollutant of each item of the instrument that affects the accuracy of our measurement. Reliability concerns the question of how much of the true score is reflected in the observed score. Thus, the reliability index of a scale item is defined as the correlation between the true score and the observed score while the square of this correlation is the reliability coefficient of the item (Raykov and Marcoulides, 2011). Note that this definition of reliability is for a scale item. This constitutes a challenge because most instruments in psychology, social sciences, and education have more than one item to measure a construct.

The conceptualization of the reliability coefficient of an instrument containing more than one item was addressed in the Spearman–Brown prophecy formula (Lord and Novick, 1968). Despite the simplicity of the Spearman–Brown prophecy formula, there are some obvious challenges in its application in a practical setting. First, the formula is defined for the entire population that we can hardly access in practice. Second, the true scores of the items are latent scores that cannot be precisely obtained in a sample study. More importantly, it is extremely difficult, if not impossible, to design parallel items for the construct of interest in educational, psychological, and social science research. This is because parallelism here means that any pair of scale items have the same true score and the same error variance (Lord and Novick, 1968). That is, parallel items measure the same true score with the same accuracy across every individual in the population, which is unrealistic in designing educational, psychological, and social science scales. For these reasons, methodologists and researchers used different methods to estimate the reliability coefficient of a scale. Two approaches to these estimations may be identified. The first approach capitalized on correlations (otherwise called coefficients of stability) either between scores of two similar forms (i.e., alternate forms) of an instrument or between scores on an instrument administered two times (i.e., test–retest with or without alternate forms). These methods suffer from errors emanating from differences in the content of the forms, confounding factors in an elapsed time of the retest coupled with practical constraints in administering a scale two times, and a lack of empirical studies on how well such correlations estimate the reliability coefficient (McDonald, 2011; Raykov and Marcoulides, 2011). Meanwhile, some researchers (e.g., Mao et al., 2017; Hunt et al., 2021) still report such correlations as evidence of scale reliability in mathematics education research. The second approach (otherwise known as the internal consistency method) estimates the reliability coefficient from scores of a single-scale administration. Statistics resulting from this approach are usually referred as to coefficients of equivalence. The coefficient alpha falls in this category.

## Coefficient alpha and the reliability coefficient

The relationship between coefficient alpha and the reliability coefficient of an instrument is far more complex than how some researchers in mathematics education make it appear. The question of how well coefficient alpha is dependable as an estimate of the reliability coefficient is subjected to assumptions that are untenable in mathematics education research. The report by Novick and Lewis (1967) is among the first attempts to address this question. Therein, they showed that coefficient alpha is a lower bound of the reliability coefficient of an instrument if there is no error correlation between any pair of its items. For a unidimensional instrument, Novick and Lewis (1967) showed that a necessary and sufficient condition for coefficient alpha to equal the reliability coefficient is essential tau-equivalence of the items. In non-technical terms, essential tau-equivalence means that each item of an instrument taps the construct of interest in the instrument with the same strength. That is, the factor loadings of the items are equal after running factor analysis to explore the factor structure of the instrument. I contend that coefficient alpha is dependable as an estimate of the reliability coefficient of an instrument provided the instrument is unidimensional with essentially tau-equivalent items and uncorrelated errors. It is important to remark that these assumptions—unidimensionality, essential tau-equivalence, and uncorrelated item errors—are practically testable using basic exploratory and/or confirmatory factor analysis (E/CFA). Furthermore, Raykov (1997) showed that essential tau-equivalence may be relaxed, in practice, if the average factor loading is high (i.e., 0.60 and above) and the instrument contains more than five items.

More importantly, if some of these assumptions are not met, the consequences on the dependability of coefficient alpha are substantial. For instance, if only tau-equivalence is violated or at least one item has a significantly different factor loading from others in the instrument coefficient alpha can grossly underestimate (up to 11%) the reliability coefficient (Raykov, 1997; Green and Yang, 2009). The situation is even worse if there is a violation of the uncorrelated errors assumption. That is, the instrument is unidimensional, and its items are essentially tau-equivalent, but some measurement errors of the items are correlated. In this case, Raykov (1997) showed that the coefficient alpha can be substantially higher than the reliability coefficient. This overestimation bias can be up to 20% as demonstrated by Green and Yang (2009). One can imagine how a coefficient alpha of 0.75 would give a false hope to researchers when the actual reliability coefficient of the instrument is 0.60. Finally, the violation of the unidimensionality assumption does not affect coefficient alpha as long as one sticks to the original interpretation of the latter as the degree of item interrelatedness (Sijtsma, 2009; McNeish, 2018). In sum, coefficient alpha may underestimate, overestimate, and only equals the reliability coefficient of a unidimensional instrument under restrictive conditions of tau-equivalence and uncorrelated errors.

## Overuse of coefficient alpha in mathematics education research

### Indiscriminate interpretation of coefficient alpha as scale reliability

The most common misuse of coefficient alpha in mathematics education research is using statistics to gauge scale reliability without paying attention to conditions under which the coefficient is trustworthy. For instance, all the articles published in the Journal for Research in Mathematics Education (JRME) between 2021 and April 2022 that reported coefficient alpha as a measure of reliability did so without any information on crucial assumptions of essential tau-equivalence and uncorrelated errors of the scale items (Battey et al., 2021; Earnest and Chandler, 2021; Lubienski et al., 2021; Santana et al., 2021). This indiscriminate use of coefficient to gauge scale reliability is not limited to published papers in JRME but widespread in papers published by other top mathematics education journals (e.g., Dowker et al., 2019; Regier and Savic, 2020; Saadati et al., 2021; Wang et al., 2022). It is common in mathematics education research articles to see statements like “The scale consisted of 4 items, and its reliability (Cronbach’s alpha) was 0.835” (Krawitz et al., 2021, p. 347) or similar wordings that carry the same meaning. I contend that such statements are true only if the researchers verify the underlying assumptions that substantiate the plausibility of coefficient alpha to serve that purpose. In the absence of such verifications, coefficient alpha offers little or no value to scale reliability other than the degree of the interrelatedness of the scale items.

### Coefficient alpha and internal consistency

The use of coefficient alpha as a measure of internal consistency of scale items is very common in mathematics education research (e.g., Widder et al., 2019; Regier and Savic, 2020; Irakleous et al., 2021; Rodríguez-Muñiz et al., 2021; Saadati et al., 2021). Using coefficient alpha in this way should ordinarily be tolerable except that some mathematics education researchers misrepresent the meaning of internal consistency. On the one hand, there are some researchers (e.g., Kop et al., 2020; Earnest and Chandler, 2021) that equate internal consistency with scale reliability. The case of these researchers is like the indiscriminate use of coefficient alpha as an estimate of scale reliability that was treated in the last section. On the other hand, some researchers equate internal consistency with the unidimensionality of an instrument. For instance, Regier and Savic (2020) wrote “[t]he Cronbach’s alpha reliability estimates of the SEPS were 0.92, 0.90, and 0.92 for Surveys 1, 2, and 3, respectively (≥0.9 is excellent), indicating that the SEPS is measuring one construct (p. 12, italics not in the original). A similar misuse of coefficient alpha can also be found on p. 510 of the article by Widder et al. (2019). I reinstate that there is a sharp contrast between the internal consistency and unidimensionality of an instrument. The former is concerned with the interrelatedness of a set of items of an instrument while the latter is a question of whether the set of items measures a single construct (Schmitt, 1996).

## Alternatives to coefficient alpha

There are several alternatives to coefficient alpha in the literature with some researchers (e.g., McNeish, 2018) claiming that more than 30 such alternatives exist. However, some of these alternatives are extensions or refinements of others (baseline versions) that address the limitations of the baseline versions. Some of the baseline alternatives to coefficient alpha are greatest lower bound (GLB) (Sijtsma, 2009), coefficient omega (ω) (McDonald, 2011), and the latent variable modeling (LVM) approach (Raykov and Marcoulides, 2016). For a unidimensional instrument with uncorrelated errors, Sijtsma (2009) claimed that the GLB is the greatest lower bound of the reliability coefficient and showed that it outperformed coefficient alpha when the essential tau-equivalence assumption is violated. Even though the greatest lower bound claim was refuted by Revelle and Zinbarg (2009) who showed that the coefficient omega is greater than the GLB, the latter remains a better estimate of the reliability coefficient than the coefficient alpha. In addition, the computation of GLB is relatively complex and not readily available in open-source statistical tools, unlike the coefficient omega that can be easily calculated using results from factor analysis. For these reasons, I will favor coefficient omega over GLB in subsequent paragraphs.

Coefficient omega is defined as a unidimensional instrument with or without correlated errors as a function of factor loadings, error variance, and covariance (Revelle and Zinbarg, 2009; McDonald, 2011). An appealing quality of coefficient omega is that the quantities upon which its function depends are readily available from the output of factor analysis in widespread statistical tools such as SPSS, STATA, R, and Mplus. Both simulated and empirical studies suggest that coefficient omega estimates the reliability coefficient of an instrument better than coefficient alpha and only equals the latter when the essential tau-equivalence assumption holds (Zinbarg et al., 2005; Revelle and Zinbarg, 2009; Dunn et al., 2014; McNeish, 2018). Some of these studies (e.g., Dunn et al., 2014; McNeish, 2018) even provide work examples that are easy to follow including programming codes and software packages for computing coefficient omega in popular statistical software and using Excel spreadsheets. These references could offer succor to mathematics education researchers seeking alternative statistics to coefficient alpha in a situation where essential tau-equivalence and uncorrelated error assumptions are violated for a unidimensional instrument.

A crucial alternative to coefficient alpha for estimating the reliability coefficient of a multidimensional instrument is the LVM approach by Raykov and Marcoulides (2016). Admittedly, there are some refinements (e.g., Zinbarg et al., 2006, 2016) of coefficient omega to suit instruments that measure more than one construct. Still, I contend that such refinements are not robust enough to violations of multiple assumptions as of the LVM approach. These assumptions include correlated errors, missing data at random or otherwise, non-normality, and non-trivial correlations between the scale constructs (Raykov et al., 2010; Raykov and Marcoulides, 2016). In addition, the LVM approach is equally dependable for estimating the reliability coefficient of unidimensional instruments under violations of coefficient alpha assumptions (Raykov, 1997). The logic of the LVM approach is like that of coefficient omega using factor analysis and model parameters to estimate the scale reliability. Empirical evidence (e.g., Raykov, 1997; Raykov and Marcoulides, 2016) shows that the LVM approach is highly robust to the violation of assumptions. It is easy to compute using popular statistical software, and Raykov and Marcoulides (2016) provided step-by-step work examples including Mplus and R codes for its computation.

## Conclusion

To conclude, I recommend the use of coefficient alpha as evidence of scale reliability only after ensuring the following restrictive analytical procedures. The first step is to check whether the instrument is unidimensional by investigating the consistency of a single-factor model of the instrument with the data using factor analysis. The second step is to confirm the essential tau-equivalence of the scale items by constraining the factor loadings to be equal and determining the fitness of the model with the data using CFA. Alternatively, one can rely on the recommendation by Raykov (1997) by checking whether the average factor loading is at least 0.60 for a unidimensional instrument with more than five items. The last step is to investigate correlated errors with some help from modification indices in the output of a CFA. If the null hypothesis of any of these procedures is not supported, then coefficient alpha is not appropriate as evidence of reliability for the scale under investigation. In this situation, the value of coefficient alpha can be high, giving false hope to researchers, or small, leading to a rejection of a reliable instrument. The remedy is to employ alternatives such as coefficient omega and the LVM approach to gauge the reliability evidence of the instruments. These alternatives are briefly discussed in the present article with references to non-technical articles where worked examples and programming codes can be found.

## Data availability statement

The original contributions presented in this study are included in the article/supplementary material, further inquiries can be directed to the corresponding author.

## Author contributions

YZ: conceptualization, methodology, formal analysis, software, data curation, investigation, visualization, and writing—original draft preparation.
